# Structured Digital Device Instruction for Older Adults With Stroke During Convalescent Rehabilitation: Prospective Feasibility Study

**DOI:** 10.2196/95768

**Published:** 2026-09-17

**Authors:** Daisuke Ito, Masayuki Dogan, Miho Nambu, Takahiro Nishiyama, Juntaro Sakazaki, Takatsugu Sato, Ayumi Watanabe, Ryota Sato, Nao Isogai, Marino Yamaguchi, Mamoru Kawakami, Kunitsugu Kondo, Michiyuki Kawakami

**Affiliations:** 1 Department of Rehabilitation Medicine, School of Medicine, Keio University, 35 Shinanomachi, Shinjuku-ku, Tokyo, 160-8582, Japan, 81 3-5843-6169, 81 3-3225-6014; 2 Department of Rehabilitation Medicine, Tokyo Bay Rehabilitation Hospital, Narashino City, Chiba, Japan

**Keywords:** stroke, rehabilitation, older adults, digital health, digital technology, feasibility, usability

## Abstract

**Background:**

Digital health technologies are increasingly used in stroke rehabilitation, but older adults with stroke may experience barriers related to cognitive, physical, motivational, and usability factors. Structured support during inpatient rehabilitation may provide an opportunity to acquire basic digital device skills before discharge.

**Objective:**

This study aimed to evaluate the feasibility of a structured digital device instruction program and the perceived usability of the integrated digital device environment among older adults with stroke during convalescent rehabilitation.

**Methods:**

This single-center prospective feasibility study enrolled older adults (≥65 years) with stroke admitted to a Japanese convalescent rehabilitation ward. Participants received a 14-day program consisting of a 7-day therapist-supported instruction phase followed by a 7-day independent practice phase. Seven predefined tasks involving a tablet computer, chat app, video calling system, and wearable device were assessed daily using a 5-point task performance scale. Perceived usability of the integrated digital device environment was assessed on day 14 using the System Usability Scale (SUS).

**Results:**

Nineteen participants (mean age 76.3, SD 6.9 years; n=9, 47% women; mean Mini-Mental State Examination score 26.1, SD 3.2) completed the 14-day program. No participant was independent across all 7 predefined digital tasks on day 1, whereas 14 (74%) achieved independence across all predefined digital tasks by day 14. Independence reached 18 (95%) for tablet and wearable device management and basic operation tasks, whereas chat app use (n=15, 79%) and video calling (n=14, 74%) remained comparatively more challenging. The mean SUS score was 57.8 (SD 15.0), indicating moderate perceived usability of the integrated digital device environment.

**Conclusions:**

This feasibility study suggests that a structured instruction and practice program can be successfully implemented in a convalescent rehabilitation ward and may facilitate the acquisition of basic digital device skills among selected older adults with stroke. These findings should be interpreted cautiously because of the single-center design, small sample size, absence of a comparator group, short follow-up period, and inclusion of relatively high-functioning participants.

## Introduction

Digital health technology (DHT), encompassing applications, software, and digital systems used to support health care delivery and health system functions [1], has garnered increasing attention in stroke rehabilitation. In recent years, diverse forms of DHT—such as virtual reality [2], robotics [3,4], telerehabilitation [5], wearable activity monitors [6], and AI [7]—have been introduced to support motor function recovery and facilitate the continuation of rehabilitation in individuals with stroke. The growing body of DHT-based intervention studies suggests that this field has become a central focus of stroke rehabilitation research [8]. Therefore, DHT is regarded as a promising next-generation intervention platform that integrates technological innovation with clinical application.

Despite this growing interest, numerous barriers hinder the clinical implementation of DHT. A systematic review in neurorehabilitation identified 5 primary factors impeding DHT integration: individual-level constraints (eg, advanced age and limited digital literacy), poor user experience, insufficient intervention content, restricted access to technology, and lack of support structures [9]. Another review focusing on poststroke populations highlighted barriers to mobile health (mHealth) access, including poor health status, low acceptance of technology, inadequate infrastructure, fragmented support systems, and time constraints on health care providers [10]. These challenges, arising from patients’ health conditions, attitudes toward technology, and complex interactions among patients, health care professionals, and devices, underscore the need for structured and comprehensive implementation strategies [10].

Given that stroke primarily affects older adults, it is also essential to consider aging-related barriers to DHT adoption. A systematic review focusing on older adults with chronic diseases identified cognitive and physical limitations, low digital literacy, and privacy concerns as key psychological and capability-related obstacles [11]. Similarly, a scoping review focusing on older adults identified 4 primary categories of age-related barriers to mHealth usability: cognitive barriers (eg, difficulty understanding and remembering procedures), motivational barriers (eg, low self-efficacy and reduced engagement), physical barriers (eg, impaired fine motor control), and perceptual barriers (eg, diminished vision or hearing) [12]. Therefore, effective implementation of DHT for older individuals with stroke requires not only technological readiness but also targeted strategies addressing cognitive, physical, and motivational constraints.

To address these multifaceted barriers, it is critical to create structured opportunities that enable users to gradually acquire the necessary digital skills. Previous studies have identified several facilitators of successful DHT adoption, including user experience, robust support systems, adequate time for onboarding, and opportunities for hands-on practice [9-11]. Japan’s convalescent rehabilitation wards represent a particularly suitable setting for such implementation. These wards allow patients to receive inpatient care for up to 180 days from the subacute phase of stroke onset, during which intensive rehabilitation is provided [13]. In addition to this extended hospitalization period, the structured environment and repetitive therapy sessions create favorable conditions for regular DHT exposure and stepwise skill acquisition. This setting provides a critical and time-limited window to establish digital competencies prior to discharge, potentially facilitating the transition to home-based and remote rehabilitation. Accordingly, the present study aimed to evaluate the feasibility and usability of a structured digital device instruction program in older adults with stroke during convalescent rehabilitation.

## Methods

### Study Design and Participants

This study was conducted as a single-center, prospective feasibility study at a Japanese convalescent rehabilitation hospital between April 2022 and March 2024. Participants were recruited using convenience sampling. Research staff periodically screened electronic medical records of newly admitted patients and invited those who met the eligibility criteria during the recruitment period. Because this was a feasibility study, no formal sample size calculation was performed. We aimed to recruit approximately 20 participants based on published methodological recommendations for pilot and feasibility studies, while also considering the feasibility of recruitment and intervention delivery within our clinical setting [14]. The inclusion criteria were as follows: (1) age ≥65 years, (2) a diagnosis of stroke, and (3) independent in self-care except bathing. The exclusion criteria were severe communication impairment due to aphasia or disturbance of consciousness, severe visual or hearing impairment, serious comorbidities (eg, heart disease or cancer), severe psychiatric disorders (eg, depression or schizophrenia), and planned discharge within 14 days. The criterion requiring independence in self-care except bathing was used to focus on patients whose functional status was compatible with anticipated home discharge and potential postdischarge DHT use, thereby allowing evaluation of the feasibility of the instructional program in the intended target population.

### Ethical Considerations

This study was conducted in accordance with the Declaration of Helsinki and was approved by the Ethics Committee of Tokyo Bay Rehabilitation Hospital (approval number 275). All participants provided written informed consent before study participation. Study data were deidentified before analysis and stored securely with access restricted to authorized study personnel. No personally identifiable information is reported in this manuscript. Participants did not receive financial compensation for participation.

### Digital Device Instruction Protocol

#### Overview

The digital device instruction protocol consisted of a 14-day program comprising a 7-day instructor-supported phase followed by a 7-day independent practice phase. Before the start of instruction, the research team preconfigured all digital devices to minimize the technical burden on participants, who were older adults undergoing rehabilitation. Specifically, the accessibility settings of the tablet PCs were customized for older adults, including increasing screen brightness, enlarging text size, and setting Japanese as the default keyboard language. In addition, a simplified photo-based instruction manual tailored to older adults—covering only essential tasks—was developed by the research team in advance. All required apps were preinstalled. User accounts were preregistered, and the wearable device was fully initialized before use. Accordingly, participants were not required to perform device setup, app installation, or login procedures. The devices used in this study were as follows: the tablet PC was an iPad (8th generation; Apple Inc), the chat app was Skype (Microsoft), the video calling system was Zoom (Zoom Video Communications), and the wearable device was the Fitbit Charge 5 (Google).

#### Instructor-Supported Phase (Days 1-7)

During the first 7 days, participants received approximately 20 minutes of individual instruction per day from rehabilitation professionals (occupational therapists and physiotherapists). Instruction covered 7 predefined digital device tasks, as summarized in Table 1. Each session followed a standardized structure. An illustrated manual developed specifically for this study was provided to each participant before each session. Participants were first observed attempting each task independently. If difficulties were encountered, support was provided in a stepwise manner, beginning with reference to the manual, followed by verbal instructions and therapist demonstration as needed. Instructions were adapted to each participant’s cognitive and motor function levels, and repetition was provided as required.

**Table 1.** Overview of the 7 digital device tasks included in the structured instruction program.

Tasks	Instructions
Tablet PC management and charging	Participants were instructed to store the tablet safely, avoid dropping it during transport or use, and connect the charging cable when needed
Tablet PC operation	Instruction covered turning the tablet on and off, unlocking the screen, and performing basic operations, including returning to the home screen and launching apps
Using chat apps	Participants were instructed to open the chat app, select a contact, and send one message daily using a preregistered study account Message content was unrestricted, allowing participants to share rehabilitation experiences or other daily activities freely
Video calling	Participants were instructed to access a Zoom video call through a chat link and to operate essential functions, including enabling the camera, turning mute on or off, and adjusting the volume
Wearable device management and charging	Participants were instructed to store the wearable device safely, connect it to the charger, and confirm battery status
Wearable device wearing	Instruction included attaching the wearable device to the wrist of the affected upper limb and ensuring proper fit and positioning
Wearable device operation	Participants were instructed to check daily step counts and interpret activity information displayed on the device screen

#### Independent Practice Phase (Days 8-14)

During the independent practice phase (days 8‐14), digital devices were lent to participants, who were instructed to perform all 7 tasks daily in their rooms. A licensed therapist visited each participant daily to confirm safe task performance. If a participant was unable to complete a task, additional instruction was provided as needed. As in the instructor-supported phase, support followed a standardized stepwise approach consisting of reference to the illustrated manual, verbal instruction, and therapist demonstration. Each visit lasted approximately 5 to 10 minutes.

Task performance was assessed using methods appropriate for each task. Tablet PC management and charging (task 1), tablet PC operation (task 2), wearable device management and charging (task 5), and wearable device operation (task 7) were evaluated through direct observation during the daily room visit. Using the chat app (task 3) was assessed by reviewing message logs sent to the principal investigator, whereas video calling (task 4) was assessed using chat app access logs together with direct observation during the call. Wearable device wearing (task 6) was assessed indirectly using step count or movement data recorded by the device management app. Because all participants engaged in daily rehabilitation, the absence of recorded step counts or movement data was interpreted as indicating that the wearable device was not worn, and the task was therefore classified as nonindependent.

### Evaluation Protocol

Each task was assessed using a 5-point ordinal scale based on daily therapist observation or device logs. The scoring system was 5 (independent without assistance), 4 (independent with reference to a manual), 3 (possible with verbal cueing), 2 (possible after modeling), and 1 (unable to perform). For this feasibility study, independence was defined as the ability to complete a task without direct human assistance. Therefore, scores of 4 and 5 were classified as indicating independence because participants were able to complete the task without direct human assistance, with the manual serving as an external reference tool that could also be used after discharge. In addition to task performance, participants’ perceived usability of the integrated digital device environment was assessed after the 14-day intervention using the System Usability Scale (SUS).

### SUS

The SUS is a widely used tool for evaluating the perceived usability of a system or product [15], consisting of 10 statements that are scored on a 5-point Likert scale (from 1 “strongly disagree” to 5 “strongly agree”). To calculate the total SUS score, the score contribution of each item is first converted to a 0 to 4 scale: for positively worded items (1, 3, 5, 7, and 9), the contribution is the selected scale point minus 1; for negatively worded items (2, 4, 6, 8, and 10), it is 5 minus the selected scale point. The sum of these converted values is then multiplied by 2.5, yielding a composite usability score ranging from 0 to 100. A higher SUS score is associated with greater usability. SUS scores were interpreted with reference to previously reported adjective ratings [16]. The SUS was used to assess participants’ perceived usability of the integrated digital device environment used throughout the study. The integrated digital device environment comprised the preconfigured tablet, chat app, video calling system, and wearable device, which participants evaluated as a single integrated environment rather than as individual devices or apps. Individual SUS items were not interpreted separately, as the SUS is designed to be evaluated as a composite score.

### Clinical Data and Assessments

Medical records were reviewed to collect general characteristics, including age, sex, type of stroke, side of paralysis, duration from stroke onset to admission, and length of hospital stay. Clinical assessments included the Mini-Mental State Examination (MMSE), Fugl-Meyer Assessment-Upper Extremity (FMA-UE), and Functional Independence Measure (FIM).

The MMSE is a questionnaire for evaluating cognitive function [17]. It consists of 11 items as follows (maximum score for each item): orientation to time (score: 5), orientation to place (score: 5), registration of 3 words (score: 3), attention and calculation (serial sevens or spelling; score: 5), recall (score: 3), naming (score: 2), repetition (score: 1), verbal comprehension (score: 3), written comprehension (score: 1), writing (score: 1), and construction (score: 1). The maximum score is 30 points, with a higher score representing greater cognitive function.

The FMA-UE score was used to measure upper extremity impairment [18]. The FMA-UE includes 30 motor function items and 3 reflex function items, scored on a 3-point ordinal scale (0=cannot perform, 1=partially performs, and 2=completely performs), with higher scores indicating better motor function (total score: 0‐66 points). The FMA-UE total score classified the severity of upper extremity paralysis, with scores ≤19 classified as severe, 20 to 47 as moderate, and ≥48 as mild [19].

The FIM is an observational evaluation tool for functional disability [20]. The FIM consists of 13 motor items and 5 cognitive items. The motor items consist of the following 4 categories: self-care (eating, grooming, bathing, upper body dressing, lower body dressing, and toileting), sphincter control (bladder management and bowel management), transfers (bed, chair, and wheelchair; toilet; and tub and shower), and locomotion (walking and wheelchair use and stairs). The cognitive items consist of 2 categories: communication (comprehension and expression) and social cognition (social interaction, problem-solving, and memory). Each item is scored on a 7-point scale ranging from 1 (total assistance or not testable) to 7 (complete independence) points. The total score ranges are 18 to 126 points, 13 to 91 points, and 5 to 35 points for the total score, motor score, and cognitive score, respectively, with a higher score representing greater functional independence.

### Statistical Analysis

Characteristics were summarized according to variable type and distribution. Categorical variables were summarized using frequencies and percentages. Task performance scores were summarized using frequencies and percentages for each score category. Clinical assessment scores were summarized using means (SDs) or medians (IQRs), as appropriate, after checking their distributions using Q-Q plots. SUS scores were summarized using the mean and SD. The number and percentage of participants achieving independence in each task and across all 7 tasks were summarized for days 1 and 14. Participants who achieved independence in all 7 tasks on day 14 were considered to have achieved mastery of digital device use.

## Results

Table 2 presents the characteristics of the participants. A total of 19 individuals were included in the analysis. No missing data were identified for participant characteristics, daily task performance scores, or SUS scores.

Task performance before and after the intervention is summarized in Tables 3 and 4. On day 1 (Table 3), no participant was independent in all 7 digital tasks. By day 14 (Table 4), independence was achieved by most participants across all predefined tasks, although video calling (task 4) and using the chat app (task 3) remained comparatively more challenging than the other tasks. Overall, 74% (14/19) of participants achieved independence across all 7 tasks. The time course of task acquisition among these 14 participants is shown in Multimedia Appendix 1.

The mean SUS score was 57.8 (SD 15.0), indicating moderate perceived usability of the integrated digital device environment (Table 4).

**Table 2.** Baseline demographic and clinical characteristics of the study participants (N=19).

Characteristics	Participants
Age (years), mean (SD)	76.3 (6.9)
Sex, n (%)	
Male	10 (53)
Female	9 (47)
Stroke type, n (%)	
Hemorrhage	4 (21)
Infarction	15 (79)
Side of paralysis, n (%)	
Right	10 (53)
Left	9 (47)
Duration from stroke onset to admission (days), median (IQR)	29 (20‐36)
Hospital stays (days), median (IQR)	125 (96‐141)
Duration from admission to study participation (days), median (IQR)	53 (34‐71)
MMSE^a^ score at admission (0‐30), mean (SD)	26.1 (3.2)
FMA-UE^b^ score at admission (0‐66), median (IQR)	42 (14‐61)
Admission FIM^c^ motor score (13-91), median (IQR)	47 (42‐56)
Admission FIM cognitive score (5-35), median (IQR)	27 (25‐30)
Admission FIM total score (18-126), median (IQR)	72 (68‐87)
Discharge FIM motor score (13-91), median (IQR)	84 (83‐87)
Discharge FIM cognitive score (5-35), median (IQR)	32 (29‐34)
Discharge FIM total score (18-126), median (IQR)	117 (112‐119)
Prior experience with digital device use, n (%)	13 (68)

a MMSE: Mini-Mental State Examination.

b FMA-UE: Fugl-Meyer Assessment-Upper Extremity.

c FIM: Functional Independence Measure.

**Table 3.** Task performance before the structured digital device instruction program (day 1), including independence in each predefined digital task among participants (N=19)^a^.

	Tablet PC management and charging^b^	Tablet PC operation^c^	Using chat apps^d^	Video calling^e^	Wearable device management and charging^b^	Wearable device wearing^c^	Wearable device operation^f^
Participant 1	1	2	1	1	2	2	1
Participant 2	2	4	4	2	2	2	3
Participant 3	2	2	2	2	2	2	2
Participant 4	2	2	2	2	2	2	2
Participant 5	2	2	2	2	2	2	2
Participant 6	3	3	3	3	3	4	3
Participant 7	2	2	2	2	2	2	2
Participant 8	2	2	3	2	3	2	2
Participant 9	3	2	2	2	2	2	3
Participant 10	2	2	1	1	1	1	2
Participant 11	3	2	3	3	3	4	3
Participant 12	3	4	3	2	3	2	3
Participant 13	5	4	3	2	4	4	2
Participant 14	3	3	3	2	3	2	2
Participant 15	4	5	5	3	5	5	5
Participant 16	2	2	1	2	2	2	2
Participant 17	3	4	3	3	3	3	3
Participant 18	5	3	3	2	4	5	2
Participant 19	2	2	2	2	2	2	2

a Independence was defined as a score of 4 or 5 (1=unable to perform; 2=possible after modeling; 3=possible with verbal cueing; 4=independent with reference to a manual; and 5=independent without assistance).

b Participants achieving independence: 3 (16%).

c Participants achieving independence: 5 (26%).

d Participants achieving independence: 2 (11%).

e Participants achieving independence: 0 (0%).

f Participants achieving independence: 1 (5%).

**Table 4.** Task performance after the structured digital device instruction program (day 14), including independence in each predefined digital task and System Usability Scale (SUS) scores for all participants (N=19)^a^.

	Tablet PC management and charging^b^	Tablet PC operation^b^	Using chat apps^c^	Video calling^d^	Wearable device management and charging^b^	Wearable device wearing^b^	Wearable device operation^e^	Overall mastery^a^^,^^d^	SUS score^f^^,^^g^
Participant 1	5	5	5	5	5	5	5	Yes	50
Participant 2	5	5	5	5	5	5	5	Yes	65
Participant 3	5	3	2	2	5	5	5	No	50
Participant 4	5	5	5	5	5	5	5	Yes	68
Participant 5	5	5	5	5	5	5	5	Yes	43
Participant 6	5	5	5	5	5	5	5	Yes	68
Participant 7	5	5	3	3	5	5	3	No	33
Participant 8	5	5	3	3	5	5	5	No	40
Participant 9	5	5	5	5	5	5	5	Yes	80
Participant 10	5	5	5	5	5	5	5	Yes	52.5
Participant 11	5	5	5	5	5	5	5	Yes	55
Participant 12	5	5	5	5	5	5	5	Yes	77.5
Participant 13	5	5	5	5	5	5	5	Yes	62.5
Participant 14	5	5	5	5	5	5	5	Yes	47.5
Participant 15	5	5	5	5	5	5	5	Yes	82.5
Participant 16	5	5	5	3	5	5	5	No	37.5
Participant 17	5	5	5	5	5	5	5	Yes	75
Participant 18	5	5	5	5	5	5	5	Yes	65
Participant 19	1	1	1	3	1	1	1	No	47.5

a Independence was defined as a score of 4 or 5 (1=unable to perform; 2=possible after modeling; 3=possible with verbal cueing; 4=independent with reference to a manual; and 5=independent without assistance).

b Participants achieving independence: 18 (95%).

c Participants achieving independence: 15 (79%).

d Participants achieving independence: 14 (74%).

e Participants achieving independence: 17 (90%).

f The mean SUS score was 57.8 (SD 15.0).

g Participants achieving independence, not applicable.

## Discussion

### Principal Results

This study investigated the feasibility and usability of a structured digital device instruction program for older adults with stroke in a convalescent rehabilitation ward. Following a 14-day protocol, most participants achieved independence across all predefined digital tasks, and the integrated digital device environment demonstrated moderate perceived usability. These findings suggest that a structured, stepwise instructional approach can be implemented in this population and highlight the importance of usability when implementing DHTs.

### Comparison With Prior Work

Most participants in this study successfully acquired digital device operation, which may be attributed to the structured learning opportunities and support system provided. In the context of poststroke rehabilitation, the implementation of DHT requires attention not only to individual characteristics such as physical and cognitive limitations but also to contextual factors, including instructional opportunities and support infrastructure [10]. Previous studies have reported that the presence or absence of support is a critical factor in DHT implementation [9], particularly emphasizing the facilitating role of active involvement by health care professionals [11]. Additionally, previous studies have shown that for older adults, providing tutorials and stepwise instructions at the initial stage of mHealth introduction is necessary to reduce learning barriers [21]. Similarly, structured digital skills training has been shown to improve digital device proficiency among older adults [22]. In this study, the instructional protocol was deliberately designed to incorporate these facilitating factors, which may have contributed to digital skill acquisition. These findings suggest that simplifying user interfaces alone is not sufficient; rather, successful adoption may require an integrated design that addresses the full learning process through both structured instruction and ongoing support. This point may be particularly important in convalescent rehabilitation, where patients have a limited yet structured opportunity to acquire digital skills before discharge. Beyond its role as an educational approach, this structured instructional protocol may inform future implementation strategies for integrating DHTs into routine convalescent rehabilitation practice. Nevertheless, video calling had the lowest mastery rate among all tasks, which is likely attributable to the procedural complexity involved. A previous study has identified complexity as a major barrier to technology use among older adults [23]. In our protocol, video calling involved multiple distinct steps—including launching a specific app, enabling microphone and camera functions, and adjusting settings—which may have posed additional challenges. Therefore, in addition to structured support, further simplification strategies, such as consolidating platforms and limiting the number of technologies used, may also be essential.

In addition to demonstrating the feasibility of the instructional protocol, the integrated digital device environment showed moderate perceived usability, as assessed by the SUS. A previous study has underscored the importance of usability—defined by ease of use and intuitive interface design—in the implementation of DHT in rehabilitation settings [10]. In older populations, incorporating age-inclusive design principles has been shown to improve digital accessibility, satisfaction, and autonomy [24]. Moreover, mobile app guidelines emphasize the importance of simplifying interactions and enlarging visual elements to accommodate age-related physical and cognitive changes [25]. In this study, usability was enhanced through preconfiguration of large text displays and the development of a minimal, image-based manual tailored to older users. These findings highlight that effective DHT implementation requires not only technical deployment but also attention to users’ subjective experience and perceived ease of use. Taken together, these findings suggest that attention to usability should be an important consideration when implementing DHT for older adults with stroke, particularly in poststroke rehabilitation settings where age-related and neurological factors may influence technology adoption.

### Clinical Implications

This study provides one of the few detailed reports of a clinically applicable protocol for implementing DHT among older adults with stroke admitted to a convalescent rehabilitation ward. Previous research has often lacked systematic use of implementation frameworks, highlighting the need for more structured implementation studies in rehabilitation contexts [10]. In contrast, our study describes a structured implementation approach, including a stepwise instructional protocol, device specifications, age-inclusive preparatory measures, and usability considerations, which may provide a practical framework for future implementation studies. The feasibility of this approach was likely enhanced by the unique characteristics of the convalescent rehabilitation ward system in Japan. The relatively long inpatient stays and structured daily schedules in this setting allowed for gradual exposure to DHT and integration of support into routine rehabilitation activities. Furthermore, as convalescent rehabilitation wards are designed to support discharge planning, skill acquisition during hospitalization may facilitate postdischarge self-management and community-based DHT use. Implementation models such as the one described in this study may provide a practical framework for future implementation efforts aimed at supporting DHT use after discharge. Future studies are needed to evaluate their effects on long-term DHT adoption and integrated community care. However, in this study, the instructional sessions were conducted outside of regular clinical hours by research staff. In addition, device preparation and preconfiguration were performed specifically for the study and may require further simplification for routine clinical implementation. Thus, practical challenges remain regarding who should deliver such support, when it should be provided, and how it can be operationalized in routine clinical workflows. Therefore, future implementation efforts must focus on developing and evaluating sustainable implementation models that can be integrated into existing clinical operations. These may include role-sharing among interdisciplinary staff, streamlined device preparation and configuration procedures, and practical tools to support DHT instruction in resource-constrained environments.

### Limitations

This study has several limitations. First, this was a single-center, single-arm feasibility study with a short duration, no comparator group, and a small sample size (n=19), which limits the ability to attribute the observed findings to the instructional program and restricts the generalizability of the results. Second, participants were required to be independent in self-care except bathing, resulting in a study population with relatively high functional independence. Accordingly, the findings may not be generalizable to stroke survivors with more severe physical or cognitive impairments. However, this eligibility criterion was intentionally adopted to evaluate the feasibility of digital device instruction in patients considered potential users of DHTs after discharge, thereby reflecting the intended target population for implementation in convalescent rehabilitation. Third, because the study assessed only short-term acquisition of digital skills and did not include a follow-up assessment, the long-term retention of these skills, actual use after discharge, and potential effects on health-related outcomes remain unclear and warrant further investigation. In addition, independence was operationally defined as task completion without direct human assistance, including completion with reference to the manual. This definition may overestimate unaided real-world autonomy because some participants still relied on the manual to complete certain tasks. Therefore, the reported independence should be interpreted as the ability to perform tasks without direct human assistance rather than complete mastery without external support. Fourth, the SUS is a subjective measure and may be influenced by cognitive function in older adults. Future research should incorporate additional objective usability measures, such as task completion time, error rates, and device use logs, to complement subjective assessments. In addition, because the SUS was originally developed to evaluate a single system, its application to the integrated digital device environment should be interpreted cautiously. Accordingly, the SUS score reflects participants’ overall perception of the integrated digital device environment rather than the usability of any individual device or app. Finally, the devices and instructional methods used in this study were specific to the intervention and context; therefore, future studies should explore the applicability of the approach using different technologies, software, or remote support models. Despite these limitations, this study presents one of the few systematically described protocols for supporting DHT adoption in convalescent rehabilitation settings, offering valuable insights from an implementation science perspective. Moving forward, the development and evaluation of long-term operational models will be essential to determine how skills acquired during inpatient rehabilitation can be maintained and applied in home environments, contributing to self-management and remote care in older adults with stroke.

### Conclusions

This study demonstrated the feasibility of implementing a structured digital device instruction program for older adults with stroke in a convalescent rehabilitation ward. Most participants achieved independence across the predefined digital tasks during the 14-day program, and the integrated digital device environment demonstrated moderate perceived usability. These findings suggest that structured instruction delivered during inpatient rehabilitation may facilitate the acquisition of basic digital competencies before discharge. However, because this was a single-center feasibility study with a small sample and no comparator group, the findings should be interpreted cautiously. Future controlled studies should evaluate the long-term retention of digital skills, postdischarge DHT use, clinical outcomes, and the scalability of this implementation approach across different rehabilitation settings.

## Supplementary material

10.2196/95768 Multimedia Appendix 1 Time course of task acquisition showing the number of days required to achieve task-specific mastery among participants who reached full independence (n=14).

## Abbreviations

DHT: digital health technology
FIM: Functional Independence Measure
FMA-UE: Fugl-Meyer Assessment-Upper Extremity
mHealth: mobile health
MMSE: Mini-Mental State Examination
SUS: System Usability Scale
