# Supplementary material for: Structured Digital Device Instruction for Older Adults With Stroke During Convalescent Rehabilitation: Prospective Feasibility Study
Source: JMIR Form Res. 2026 Sep 17;10:e95768. doi: 10.2196/95768 (PMC13585364; doi:10.2196/95768)
Supplement: Supplementary file 1 [file formative-v10-e95768-s001.doc]

| Multimedia Appendix 1. Daily Task Achievement in Participants who Achieved Independence (n = 14).  To examine the time course of task acquisition, we summarized the number of days required to achieve task-specific mastery among participants who achieved independence in all seven tasks (n = 14). Independence in tablet PC management and charging was achieved by day 6, tablet PC operation by day 4, using chat apps by day 8, and video calling by day 13. For wearable device tasks, independence in management and charging was achieved by day 5, and independence in both wearing and operation was achieved by day 7. | | | | | | | | |
| --- | --- | --- | --- | --- | --- | --- | --- | --- |
| Day of Achievement | Tablet PC | | | |  | Wearable device | | |
|  | Management/charging | Operation | Using chat apps | Video calling |  | Management/charging | Wearing | Operation |
| Day 1 | 3 (21%) | 5 (36%) | 2 (14%) | 0 (0%) |  | 3 (21%) | 5 (36%) | 1 (7%) |
| Day 2 | 11 (79%) | 9 (64%) | 2 (14%) | 1 (7%) |  | 9 (64%) | 10 (71%) | 4 (29%) |
| Day 3 | 13 (93%) | 12 (86%) | 7 (50%) | 5 (36%) |  | 12 (86%) | 11 (79%) | 9 (64%) |
| Day 4 | 12 (86%) | 14 (100%) | 8 (57%) | 5 (36%) |  | 13 (93%) | 12 (86%) | 7 (50%) |
| Day 5 | 13 (93%) | 14 (100%) | 9 (64%) | 9 (64%) |  | 14 (100%) | 12 (86%) | 10 (71%) |
| Day 6 | 14 (100%) | 14 (100%) | 7 (50%) | 8 (57%) |  | 14 (100%) | 13 (93%) | 12 (86%) |
| Day 7 | 13 (93%) | 13 (93%) | 9 (64%) | 8 (57%) |  | 13 (93%) | 14 (100%) | 14 (100%) |
| Day 8 | 14 (100%) | 14 (100%) | 14 (100%) | 9 (64%) |  | 13 (93%) | 13 (93%) | 12 (86%) |
| Day 9 | 13 (93%) | 14 (100%) | 14 (100%) | 9 (64%) |  | 14 (100%) | 14 (100%) | 13 (93%) |
| Day 10 | 14 (100%) | 14 (100%) | 13 (93%) | 11 (79%) |  | 14 (100%) | 14 (100%) | 14 (100%) |
| Day 11 | 14 (100%) | 14 (100%) | 13 (93%) | 12 (86%) |  | 14 (100%) | 13 (93%) | 13 (93%) |
| Day 12 | 14 (100%) | 14 (100%) | 13 (93%) | 12 (86%) |  | 14 (100%) | 14 (100%) | 14 (100%) |
| Day 13 | 14 (100%) | 14 (100%) | 13 (93%) | 14 (100%) |  | 14 (100%) | 14 (100%) | 14 (100%) |
| Day 14 | 14 (100%) | 14 (100%) | 14 (100%) | 14 (100%) |  | 14 (100%) | 14 (100%) | 14 (100%) |
